# Reduced Intracellular Drug Accumulation in Drug-Resistant Leukemia Cells is Not Only Solely Due to MDR-Mediated Efflux but also to Decreased Uptake

**DOI:** 10.3389/fonc.2014.00306

**Published:** 2014-10-31

**Authors:** Angela Oliveira Pisco, Dean Andrew Jackson, Sui Huang

**Affiliations:** ^1^Institute for Systems Biology, Seattle, WA, USA; ^2^Faculty of Life Sciences, University of Manchester, Manchester, UK; ^3^Department of Biological Sciences, University of Calgary, Calgary, AB, Canada

**Keywords:** endocytosis-related multi-drug resistance, ABC transporters dependent drug resistance, cellular responses to anticancer drugs, novel mechanism of multi-drug resistance, acute myeloid leukemia model

## Abstract

Expression of ABC family transporter proteins that promote drug efflux from cancer cells is a widely observed mechanism of multi-drug resistance of cancer cells. Cell adaptation in long-term culture of HL60 leukemic cells in the presence of chemotherapy leads to induction and maintenance of the ABC transporters expression, preventing further accumulation of drugs. However, we found that decreased accumulation of drugs and fluorescent dyes also contributed by a reduced uptake by the resistant cells. Confocal time-lapse microscopy and flow cytometry revealed that fluid-phase endocytosis was diminished in drug-resistant cells compared to drug-sensitive cells. Drug uptake was increased by insulin co-treatment when cells were grown in methylcellulose and monitored under the microscope, but not when cultured in suspension. We propose that multi-drug resistance is not only solely achieved by enhanced efflux capacity but also by supressed intake of the drug, offering an alternative target to overcome drug resistance or potentiate chemotherapy.

## Introduction

**Figure 1:**
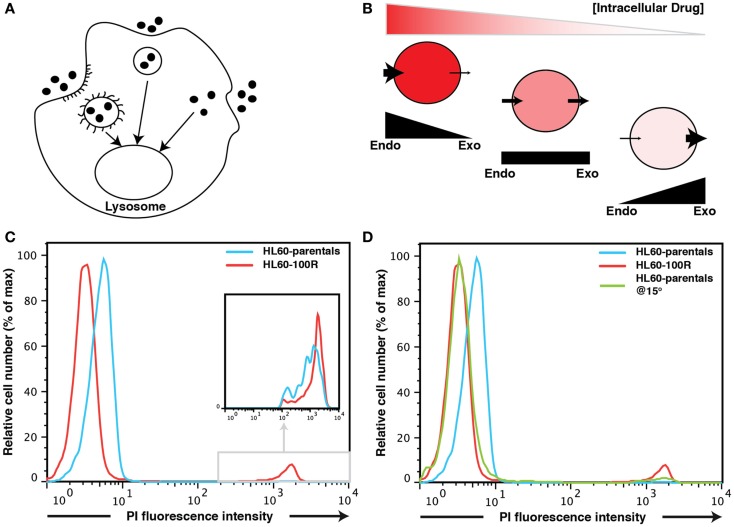
**Fluid-phase endocytosis in HL60-parentals and HL60-100R**. **(A)** Schematic representation of fluid-phase endocytosis. Pinocytotic vesicles fuse with lysosomes that hydrolyze the pinocytosed material. **(B)** Unlike receptor-mediated endocytosis, pinocytosis is non-specific to the substances that it transports. The cellular net balance between endocytosis (endo) and exocytosis (exo) regulates the accumulation of drug inside the cells. **(C)** PI accumulation in HL60-parentals and HL60-100R. Dye accumulation was measured using flow cytometry. PI was added just before flow cytometry analysis of HL60-parental cells and HL60-100R. The cells, initially at room temperature, where kept on ice until analysis. The differential PI accumulation is only seen on the left peak (cells that take up PI via endocytosis), not on the small far right peak (dead cells). The second peak is barely noticeable for HL60-parentals but zooming reveals the overlap (inset). **(D)** HL60-parentals and HL60-100R were incubated for 15 min at 15°C with PI. As control, HL60-parentals and HL60-100R cells were kept in the incubator and PI was added just as in **(C)**. Results in **(D)** are representative of *n* = 3 biological replicates. The results in **(C)** were consistently observed every time an analysis was performed.

Endocytosis is used by all cells to take up solutes from their surroundings (1–6). The process of endocytosis can be energy-dependent or independent and contributes to the cells’ interaction with their environment. Fluid-phase endocytosis (or macropinocytosis) can be defined as the uptake of extra-cellular medium into cells by vesicular endocytosis (3, 7–12) (Figure 1A) and is the entry route for non-selective endocytosis of macromolecules (1, 5, 9, 13–15). In fluid-phase endocytosis, the uptake of the extra-cellular fluid is proportional to the concentration of molecules outside the cell (3, 16–19). In contrast, internalization of hormones and nutrients, as well as toxins and pathogens, typically occurs via receptor-mediated endocytosis. Some signaling pathways are controlled by differential endocytosis, which mediates recycling and ensues degradation of cell surface receptors (3, 18, 20, 21). Moreover, the regulation of endocytosis can also control the extent of drug delivery to a cell and disruptions of this process may have consequences in disease development (22–26).

Because pharmacological drugs are developed through a lengthy optimization processes that prioritizes cell permeability, it is commonly assumed that when they are finally approved, they can cross the cell membrane easily (2, 3, 22, 24, 26–28). Therefore, the lack of accumulation of drug molecules in drug-resistant cancer cells has been explained by their capacity to eject drugs (3, 7, 10, 20, 29–31). In this perspective, a drug-resistant cell whose resistance is conferred by the overexpression of a member of the ABC transporter family, such as MDR1 (9, 13–15, 23), accumulates a lesser amount of drug inside the cell because the transporters efficiently carry the drug out of the cell (23). This phenomenon has been established and validated many times (8, 11, 12, 16, 17, 19). A straightforward approach of blocking ABC transporters activity to counter drug resistance in cancer had focused on using competitive inhibitors that bind to the transporter with at least the same affinity as the drug, thereby preventing cellular detoxification and leading to intracellular drug accumulation (1–3, 27, 28). What this model neglects is how the drug enters the cell in the first place: the net intracellular concentration of a drug is a balance between its accumulation due to the uptake and its clearance by the transporter-facilitated efflux (Figure 1B). The current model assumes that drug uptake is constant because the influx of the drug into the cell is taken for granted. However, drug resistance may also be a consequence of reduced endocytosis/influx of cytotoxic drugs (3, 20).

Here, we ask whether modulation of intake can also contribute to the acquisition of drug resistance phenotype following chemotherapy treatment. We recently showed that inhibition of drug accumulation as a resistance mechanism in HL60 cells is not simply the result of a Darwinian selection for cells with mutations that endow them with capacity of high rate of drug ejection, but instead, reflects an active, drug induced cellular response conferred by rapid up-regulation of expression of MDR1 (22, 23). Interestingly, we found that propidium iodide (PI) and doxorubicin uptake differed in HL60-treatment-naïve (HL60-parental) versus HL60-vincristine resistant cells. By studying HL60-parental cells and HL60 cells resistant to 10 nM of vincristine (HL60-10R) and to 100 nM of vincristine (HL60-100R), we were able to show that a reduction in fluid-phase endocytosis appeared at the late stage of cellular adaptation to the drug and was necessary for a stable MDR phenotype. While verapamil, a well-known inhibitor of MDR1, was not able to re-establish the drug accumulation balance observed in non-resistant cells, two endocytosis facilitating agents, PMA and insulin, promoted drug accumulation in the resistant cells. Taken together, our results suggest that a model in which expression of ABC transporters prevent drug accumulation in the resistant cell by improving efflux must be extended by considering the other side of the equation, the suppression of drug influx by active reduction of endocytosis.

## Results

### HL60-100R cells do not accumulate PI to the same extent as HL60-parental cells

Propidium iodide flow cytometric assay is routinely used to evaluate population viability by discriminating live (dye negative) from dead (dye positive) cells in many different types of cells (8, 9, 11, 12, 24, 26, 33). It is commonly assumed that viable cells are not permeable to PI and that PI labeled cells represent dead cells. PI binds to DNA by intercalating between the bases with a stoichiometry of one dye per 4–5 bp of DNA (1, 3, 5, 6) and once the dye is bound to nucleic acids, its fluorescence is enhanced 20- to 30-fold (13, 18, 23, 34). However, the use of this dye as a live/dead discriminant must consider that PI actually can enter live cells through *fluid-phase endocytosis* and has in fact been used as a marker for this endocytic route (8, 9, 12, 17, 19, 24, 26, 35, 36).

After a short (∼5 min) incubation time, HL60-parental cells were slightly but consistently more permeable (hence brighter) to the dye than their resistant counterparts as evidence by flow cytometry of PI treated cells (Figure 1C). The difference in the PI-uptake profile between HL60-100R and HL60-parentals was consistently observed. Without gating out the dead cells (Figure 1C), one can observe that dead cells are about three-logs brighter than live cells. This second peak on the far right of the fluorescence intensity axis of the flow cytometry histograms was consistent for all cell lines. Thus, even if a 5 min incubation at room temperature is sufficient to load the cells, PI can still be used for live/dead discrimination as the fluorescence intensity given by dead cells is by orders of magnitude higher than the baseline signal on the left of the fluorescence intensity axis that is due to endocytosis.

But why is the baseline of PI fluorescence in viable cells higher in HL60-parental cells than in HL60-100R cells? There are two possible explanations. First, PI may be a substrate for MDR1; in that situation, the resistant HL60-100R cells will have lower signal for PI because these cells pump the dye out more efficiently. Second, it might be that conversely, the HL60-100R cells have lower basal endocytosis for PI than the HL60-parental cells. This would also explain why we observe the differences in such a short period of time.

The most effective non-invasive method to inhibit fluid-phase endocytosis is incubation at low temperatures (bellow 20°C) (7, 8, 10–12). To investigate if the observed difference between PI signal could be due to reduced endocytosis in the resistant cells we incubated HL60-parental cells and HL60-100R cells at 15°C for 15′ with PI. As a control we used cells incubated at 37°C to which PI was added only before the experiment (Figure 1D). When parental cells were incubated at lower temperatures, their accumulation of PI shifts to the range of HL60-100R. This suggests that it is more likely that the reduced fluorescence is due to reduced uptake, rather than a more efficient expulsion of PI.

### PI accumulates in live HL60-parental and HL60-100R cells

**Figure 2:**
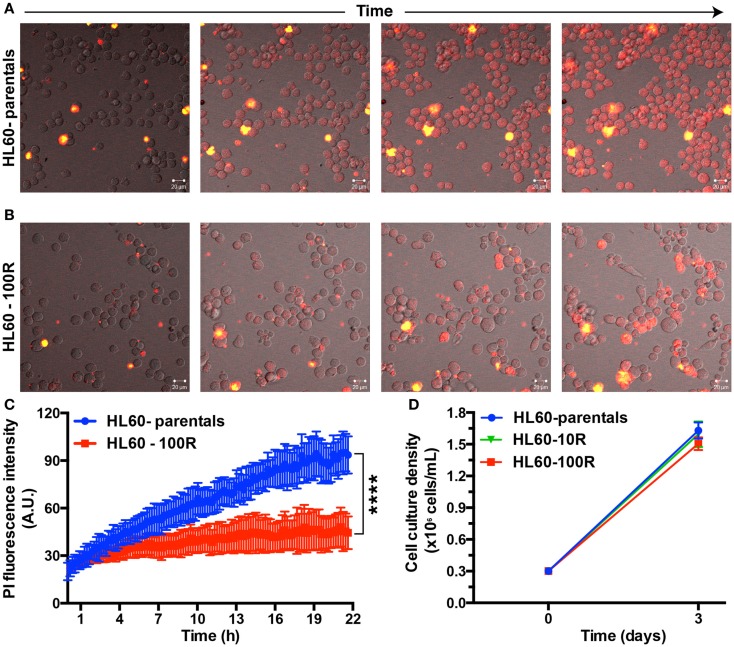
**Propidium iodide endocytosis in HL60-parental and HL60-100R cells**. **(A,B)** Snapshot of the cell population after different incubation times with PI. **(C)** Quantification of PI fluid-phase endocytosis in HL60-parentals (blue) and HL60-100R (red). Each data point represents the mean ± SD of *n* = 10 individual cells. ****p-Value < 0.0001 for time >8 h. **(D)** Growth curve for HL60-parental, HL60-10R, and HL60-100R cells. *p*-value was calculated by two-way ANOVA.

To show that PI indeed enters cells at different rates we followed HL60-parental and HL60-100R cells over time using time-lapse microscopy in cells seeded on a glass bottom dish and kept at 37°C, 5% CO_2_ (Figure 2). HL60-parental cells accumulate PI much faster than HL60-100R (Figure 2A). PI did not interfere with cellular viability, because the cells continue to proliferate throughout the entire experiment. HL60-100R reached their maximum uptake in about 8 h, after which the intensity of intracellular PI remained constant (Figure 2B). Quantitative analyses of the images revealed that the accumulation of PI was significantly different between HL60-parentals and HL60-100R (Figure 2C), supporting the qualitative observations. Moreover, the observed differences of drug accumulation are not due to differential growth rates, as sensitive and resistant cells show an identical proliferation profile (Figure 2D).

### Reduction of endocytic activity occurs at the latter stage in the establishment of resistance

The different rates of PI accumulation in the naïve and the resistant HL60 cells are not only visible in HL60-100R but also in the HL60-10R cells that were adapted to lower doses of vincristine. To determine when the phenotype of reduced endocytosis appears during the adaptation process we increased the concentration of the drug in a parallel culture of HL60-10R, from 10 to 100 nM vincristine (HL60-10R- >100R cells) and compared it to a regular culture of HL60-10R where the vincristine challenge was not increased. The kinetics of the adaptation of HL60-10R cells to this higher amount of drug was monitored in a detailed time course over the first 12 days of treatment. The data in Figure S1 in Supplementary Material suggests that the magnitude of the shift of PI accumulation depends on the degree of resistance (Figure S1A in Supplementary Material): after a period of 12 days HL60-10R cells accumulated less PI than HL60-parental cells and HL60-100R cells (resistant to higher amounts of drug) accumulated even less dye than HL60-10R cells. For the first 9 days, there were no changes (Figures S1A–C in Supplementary Material): HL60-10R and HL60-10R- >100R showed a complete overlap in PI retention. However, at day 12, we observed that the cells in the HL60-10R- >100R arm no longer accumulated as much PI as HL60-10R (Figure S1D in Supplementary Material).

A semi-quantitative analysis of the ratio of the mean of each peak relatively to the parental cells over time demonstrated the trend of PI uptake to decrease in the HL60-10R- >100R culture during its adaptation to a higher drug concentration (Figure S1E in Supplementary Material): in 12 days, the relative mean of PI signal changed by >30%, from a value close to HL60-parentals to a value close to that of HL60-100R. The relative mean of the PI signal for HL60-10R did not change in the same time interval. Although the relative mean of the PI signal in HL60-100R slightly decreased, the change was <10%. After ~20 days, HL60-10R- >100R cells were resistant to 100 nM of vincristine (viability >95%) and the PI intensity peak was identical to the one observed for HL60-100R.

### Fluid-phase endocytosis is blocked in HL60-100R

**Figure 3:**
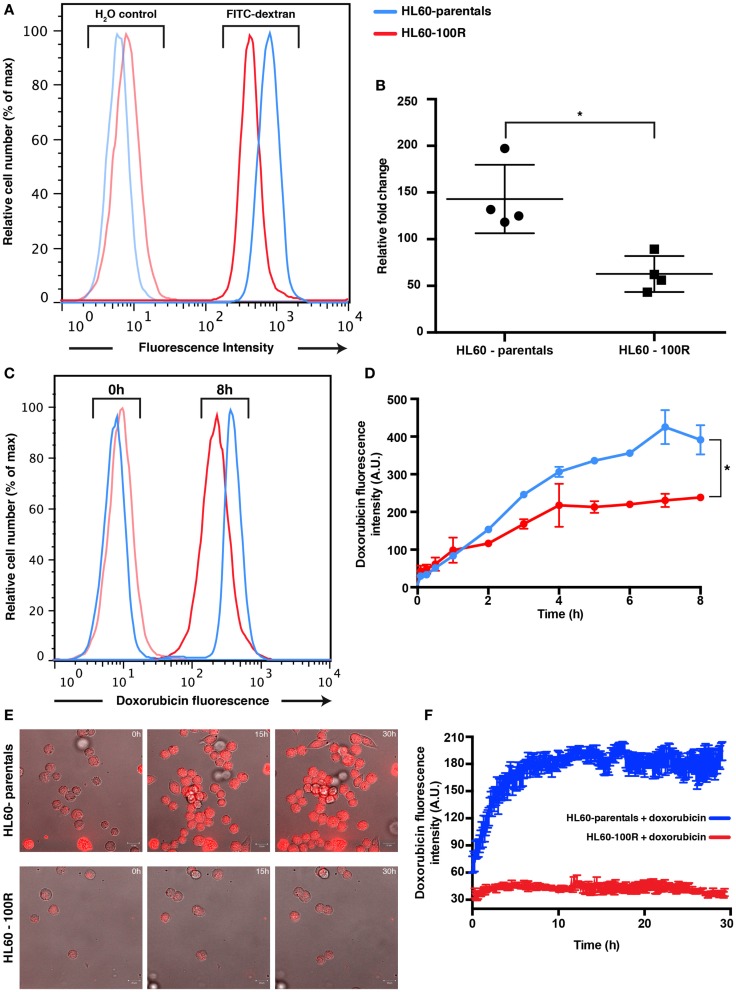
**Parental and resistance cells have different uptake capabilities**. **(A)** Reduced accumulation of FITC-dextran in HL60-100R. Cells were incubated with 1 mg/mL of FITC-dextran for 2 h at 37°C and the accumulation was measured using flow cytometry. **(B)** The mean of the accumulation peak measured by flow cytometry for *n* = 4 independent biological replicates was normalized to the respective H_2_O control. Scatter plot shows the data together with mean and SD. The two data sets are significantly different (*p-value = 0.0286 < 0.05). *p*-value calculated by unpaired non-parametric Mann–Whitney *U*-test. **(C)** Kinetics of doxorubicin uptake by HL60-parental and HL60-100R cells. Cells were incubated with 5 μM of doxorubicin at 37°C. After various time intervals, the cells were washed in HBSS/5% FBS and analyzed in flow cytometry. **(D)** Quantification of the flow cytometry data. Each data point represent the mean ± SD of *n* = 2 independent biological replicates. The data sets are significantly different (*p-value = 0.0320 < 0.05). *p*-Value calculated by Wilcoxon matched-pairs signed rank test. **(E)** *In vitro* confocal images of subcellular doxorubicin fluorescence distribution in HL60-parental and HL60-100R cells and respective quantification **(F)** for *n* = 10 cells.

To determine if the reduced accumulation of PI was due to a generic down-regulation of non-receptor-mediated fluid-phase endocytosis, we also measured uptake of FITC-Dextran after 2 h of incubation at 37°C in HL60-parentals and HL60-100R (Figures S3A,B in Supplementary Material). FITC-Dextran is a well-established marker used to assess fluid-phase endocytosis status (1, 9, 14, 15). Accumulation of the fluid-phase endocytosis marker was significantly higher in HL60-parentals than in HL60-100R (Figure 3A). After 2 h of incubation with 1 mg/mL of FITC-Dextran at 37°C, we observed approximately threefold lower uptake of FITC-Dextran by HL60-100R cells when compared to their sensitive counterparts (Figure 3B). Because the auto-fluorescent background is higher in HL60-100R than in HL60-parentals (Figure 3A; H_2_O control), we normalized each independent replicate to its respective control, and calculated the relative fold change. Although the SD was higher for HL60-parentals, the average of the replicates indicated that the two cell lines were significantly different regarding their rate of fluid-phase endocytosis.

### Doxorubicin uptake is diminished in resistant cell lines

Based on PI and FITC-Dextran as fluid-phase endocytosis markers, we have shown that endocytosis is lower in the resistant cell lines. Yet, a reduction of endocytosis *per se* does not automatically imply increased drug resistance. Thus, we next examined the functional consequences of this reduced endocytosis by following the uptake of doxorubicin, a well-established chemotherapeutic drug known to be a substrate for MDR1. Because doxorubicin is fluorescent, we can also use the drug fluorescence signal as the direct reporter for intracellular drug accumulation. A saturating (supra-maximal) concentration of doxorubicin was used to ensure sufficient intake that could override the contribution of MDR1 activity.

Flow cytometry analysis of doxorubicin uptake up to 8 h of incubation of cells with drug indicated a twofold higher uptake in the HL60-parental cells compared to the HL60-100R cells during the incubation period (Figures 3C,D). The amount of drug accumulated in the HL60-100R cells reached a plateau about 4 h after the treatment start. Since the control cell line continued to accumulate, the drug was not degraded during the course of the experiment. Taken together, these experiments indicate that the down-regulation of drug fluid-phase endocytosis in HL60-resistant cells had functional consequences. The reduced intracellular accumulation of chemotherapeutical drugs, such as doxorubicin, may suggest that perhaps the MDR phenotype is not only achieved by increase efflux but is also accompanied by a reduction in endocytosis. Confocal microscopy measurement corroborated the results obtained with flow cytometry (Figures 3E,F). The net uptake of doxorubicin in HL60-parental cells was much faster than in the resistant cells and after 4 h the accumulation remain stable, with approximately threefold difference in the intensity between HL60-parental (blue) and HL60-100R cells (red). The observation that MDR cells do not accumulate doxorubicin to the same extent as parental cells is also corroborated by previous studies (3, 16).

### PI is not a substrate of MDR1

**Figure 4:**
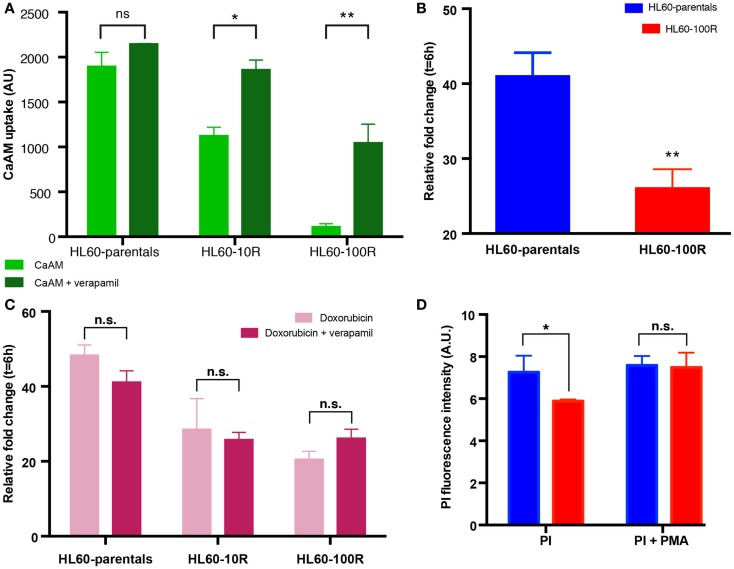
**Sensitive cells accumulate more chemotherapeutical drug**. **(A)** In the presence of verapamil, the retention of CaAM is significantly higher in the resistant cell lines, consistent with an expectable significant reduction in the ABC transporters activity. **(B)** Cells were incubated with 5 μM of doxorubicin and 10 μM of verapamil at 37°C. Co-administration of doxorubicin with verapamil is not sufficient to significantly increase the uptake of doxorubicin in HL60-100R. **(C)** Doxorubicin uptake is similar when the drug is administered alone or in combination with verapamil for all cell lines. **(D)** Effect of PMA on HL-60 cells. Quantification of the flow cytometry data of HL60-parental and HL60-100R cells incubated with 100 nM of PMA at 37°C for 15 min. Each data point represent the mean ± SD of *n* = 3 independent biological replicates. The data sets are significantly different (*p-value = 0.0279 < 0.05). *p*-value calculated by two-way ANOVA.

To further support the notion that suppression of PI accumulation was due to down-regulation of endocytosis, rather than MDR1-mediated efflux, we incubated HL60-parentals and HL60-100R with verapamil and doxorubicin. Verapamil is a potent inhibitor of ABC transporter activity (22, 27, 28). Incubation of all cell lines with verapamil, prior to CaAM efflux assay, resulted in a significant increase in dye accumulation (Figure 4A). If the differences in the accumulation of doxorubicin between HL60-parental and HL60-100R cells were solely due to drug efflux, verapamil should abrogate these differences. We observed that in the presence of verapamil the accumulation of doxorubicin was increased in the HL60-100R cells (Figures 4B,C). Nevertheless, this response did not suffice for the cells to reach the levels seen in the HL60-parental cells and the difference in the net accumulation further increased over time.

### PMA can rescue the phenotype

Since verapamil was not able to re-establish the net accumulation by blocking the efflux, we next explored the increase in uptake stimulated by phorbol-12-myristate-13-acetate (PMA), a drug known to promote endocytosis (7, 10, 25, 37, 38). HL60-parental and HL60-100R cells were co-treated with PMA and PI. While baseline PI accumulation in HL60-100R was significantly lower than in HL60-parental cells, in the presence of PMA the accumulation was about the same level in both cell lines (Figure 4D), suggesting that the uptake machinery in the HL60-100R was still intact but suppressed. This result has practical importance, given that the reduced endocytosis might be directly linked to the failure of MDR1 inhibitor drugs in clinical trials.

### Insulin does not stimulate endocytosis equally in all experimental conditions

**Figure 5:**
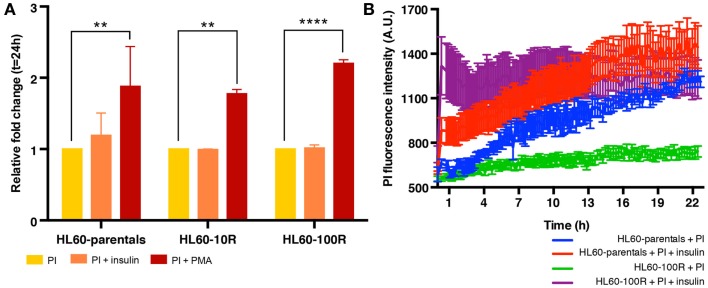
**Insulin does not stimulate endocytosis equally in all experimental conditions**. **(A)** In suspension culture conditions, PI endocytosis is stimulated by PMA but not by insulin. **(B)** Quantification of fluid-phase endocytosis of PI accumulation in HL60-parentals (blue) and HL60-100R (green) and in insulin co-treated HL60-parentals (red) and HL60-100R (purple). Each data point represent the mean ± SD of *n* = 10 individual cells.

Finally, we also tested the effect of insulin on drug uptake because stimulation of fluid-phase endocytosis by insulin is a well-established phenomenon (9, 14, 15, 33, 39), which fuels an on-going controversy in cancer therapy, as to whether or not insulin potentiation therapy (IPT) has benefits in chemotherapy outcome (6, 16, 40). Administration of insulin did not grossly affect population growth and viability of HL60 cells, permitting us to ask if co-administering PI and insulin would result in higher uptake of PI in drug-resistant HL60 cells. Co-treatment of cells with insulin and PI in regular growth medium, did not show any effect on PI uptake in the resistant cell lines (Figure 5A) – as opposed to treatment with PMA. However, when the cells were in methylcellulose (used to hold cells under the microscope), insulin administration resulted in an increase of PI accumulation in both cell lines (Figures 5B; Figure S2 in Supplementary Material). HL60-100R cells (purple) exhibited a more pronounced response with a drastic accumulation of PI, reaching the plateau almost immediately. HL60-parental cells (red) continued to uptake PI for the entire duration of the experiment. Thus, insulin can promote PI uptake but only when in methylcellulose culture raising the baseline endocytosis in naive cells and more drastically, overcoming the “adaptive” reduction of this process in the resistant HL60 cells. The reason for the distinct effect of insulin in liquid adherent cell culture vs. suspension is still unknown.

## Discussion

Development of multi-drug resistance is a chief reason for failure of chemotherapy. Here, we show that suppression of fluid-phase endocytosis was directly linked to long-term drug resistance in HL60 acute myeloid leukemic cells. This actively regulated cellular process might be associated with reduced clinical effectiveness of cancer drugs. Our results are in line with previously reported data showing defective uptake of cisplatin in resistant tumor cells (27, 28).

We excluded the possibility that PI, used here to demonstrate the contribution of uptake in the net accumulation of drugs in the cell, is a substrate for MDR1, and corroborated its use as a marker for fluid-phase endocytosis. This report neither does suggest that fluid-phase endocytosis is the sole or major mechanism of drug resistance nor does it even exclude the contribution of receptor-mediated endocytosis or differential cellular permeability. Further exploration is needed to uncover the various mechanisms of uptake and how they contribute to cellular homeostasis (25, 37, 38). Comparison of changes in cellular permeability and membrane transcytosis between parental and resistant cells would still need to be investigated due to the non-specific nature of evaluating fluid-phase endocytosis.

The study of endocytosis regulators, such as PIKfyve (33, 39) and Dynamin-2 (6, 40), which have been previously explored, will also help with future investigations. Recent reports suggest that all cellular transport might be in some way carrier-mediated and that passive diffusion is negligible (34, 41, 42). If true, this would open a new line of research toward the identification of the carriers involved in fluid-phase endocytosis or other uptake processes as a pharmacological means to overcome resistance, which has not been achieved using drugs that block ABC transporters alone (35, 36, 43).

A previous study of cancer resistance has implicated reduced endocytosis in multi-drug resistance (4, 44) but this present report establishes a direct link between long-term cellular (non-genetic) “adaptation” to drugs and fluid-phase endocytosis suggesting that the reduced net uptake of drug might be just one step in the multi-step, non-genetic phenotype change that sensitive cells undergo in response to chemotherapy to cope with toxicity (21, 23) before resistance becomes fixed through selection of adapted cells.

In summary, the present study demonstrates that a drug-uptake mechanism, possibly fluid-phase endocytosis, is not active at the same level in parental and resistant cells. The homeostatic regulation assured by the endocytosis–exocytosis cycle is crucial for normal cellular behavior, which might be the reason why disruption in this balance is often linked to disease (25, 45). The model of MDR that is based on reduced drug accumulation owing to ABC transporters alone cannot explain the present findings wherein cancer cells suppress drug accumulation to below the baseline of non-resistant cells. Here, we propose a revision to this model: therapy-stressed cells, which actively induce MDR1 expression (9, 23) as an evolved response for the detoxification in adverse environments, also induce, with some delay, a protective cell state in which entry of toxins is blocked. A more detailed, mechanistic study of the latter is needed to fully understand the role of cellular endocytosis in health and disease and for the development of new drugs that can counter the multifaceted biology of drug resistance.

## Materials and Methods

### Cell culture

HL60 cells were cultured in Iscove’s Modified Dulbecco’s Medium (IMDM, Invitrogen) supplemented with 20% fetal bovine serum (FBS, Sigma), 1% l-glutamine, penicillin (100 U/mL, Invitrogen), and streptomycin (100 mg/mL, Invitrogen). Cell number was monitored daily and culture was maintained at a density of 2 × 10^5^–2 × 10^6^ cells/mL. Cell number was monitored daily using a BioRad automatic cell counter and viability was assessed by trypan blue exclusion (0.4% supplied with the cell counter slides and added to the cells in a 1:1 v/v ratio according to manufacture’s instructions). HL60-resistant cells were supplemented with the respective concentration (10 or 100 nM) of vincristine (in H_2_O, Sigma). The cells were passaged every 2–3 days and drug was added freshly each time.

### Flow cytometry

Propidium iodide (Life Technologies) was added to suspension cells in a concentration of 5 μg/mL just before analysis unless indicated otherwise. To measure fluid-phase uptake using fluorescein isothiocyanate (FITC)-dextran, 1 × 10^5^ cells were washed in Hank’s balanced salt solution (HBSS)/5% FBS and then incubated with or without 1 mg/mL of FITC-dextran (Sigma, average mol. wt. 70,000) in fresh medium at 37°C for 2 h. Doxorubicin (Dox), verapamil (Ver), and PMA were all acquired from Sigma in powder form and dissolved in DMSO (Sigma). Calcein AM (CaAM, 1 mg/mL solution in anhydrous DMSO) was acquired from Life Technologies. Flow cytometry analyses were performed on a BD FACSCalibur cell cytometer and flow cytometry data were analyzed using FlowJo software (Tree Star). When quantification of data is presented, namely, mean values of the peak intensity, those were obtain by calculating the mean value of the distribution using FlowJo.

### Microscopy and data analysis

HL60 cells at a density of 1 × 10^5^ cells/mL were seeded for 30 min in a 35 mm four-compartments glass bottom dishes (CELLview™, Greiner Bio One). To each compartment we added 600 μL of fresh medium with methylcellulose (MethoCult™ H4100, StemCell Technologies) in a ratio 4:1. When adding PI, doxorubicin, or insulin or any combination, the drugs were first added to fresh medium and only then the mixture was added to methylcellulose. As controls we used cells in dye-free medium. Cells were imaged with a Zeiss LSM 510-Exciter (Fluar 20x/0.75 UV objective) using a Pecon incubator XL (37°C, 5% CO_2_) and the images were analyzed using LSM Aim Software. PI was excited with a 514 nm argon ion laser and detected with a 560 nm long-pass filter. Doxorubicin was excited with a 488 nm argon ion laser and detected with a 560 nm long-pass filter. For quantification, we used CellTracker version 6.0 software [DTI Beacon Project, University of Manchester (5, 32)] to mark cell boundaries of a single cell captured in consecutive time course microscopy images. The program measured average fluorescence intensity for total cell area and output that value, along with the corresponding time, into a comma-separated values (csv) file.

### Statistical analysis

The statistical tests were performed using GraphPad Prism version 6.00 for MacOS, GraphPad Software, La Jolla, CA, USA, www.graphpad.com.

## Conflict of Interest Statement

The authors declare that the research was conducted in the absence of any commercial or financial relationships that could be construed as a potential conflict of interest.

## Supplementary Material

The Supplementary Material for this article can be found online at http://www.frontiersin.org/Journal/10.3389/fonc.2014.00306/abstract
