# Supplementary figures and images for: Reduced Intracellular Drug Accumulation in Drug-Resistant Leukemia Cells is Not Only Solely Due to MDR-Mediated Efflux but also to Decreased Uptake
Source: Front Oncol. 2014 Oct 31;4:306. doi: 10.3389/fonc.2014.00306 (PMC4215691; doi:10.3389/fonc.2014.00306)

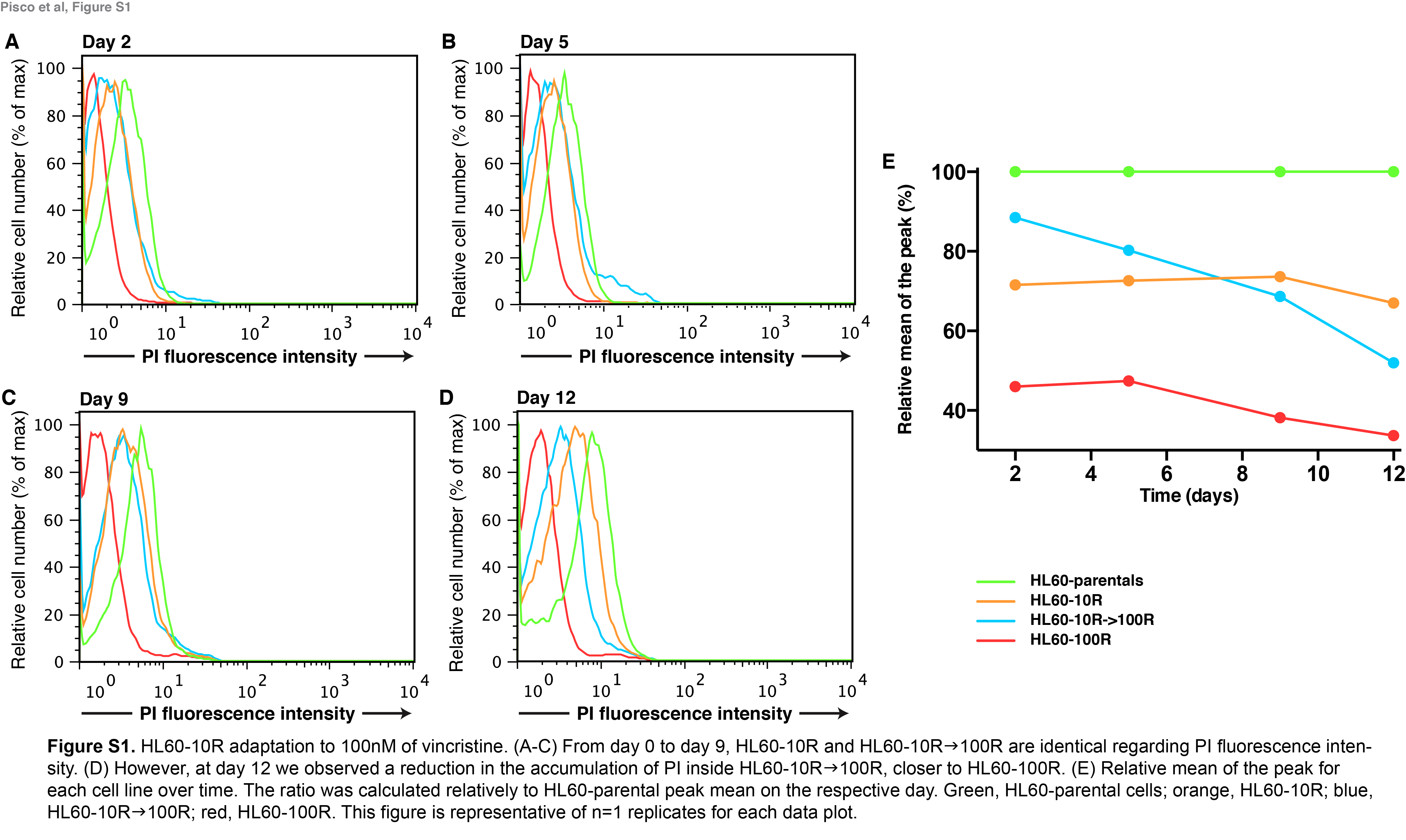

Supplement: Supplementary file 1 [file Image_1.TIF]

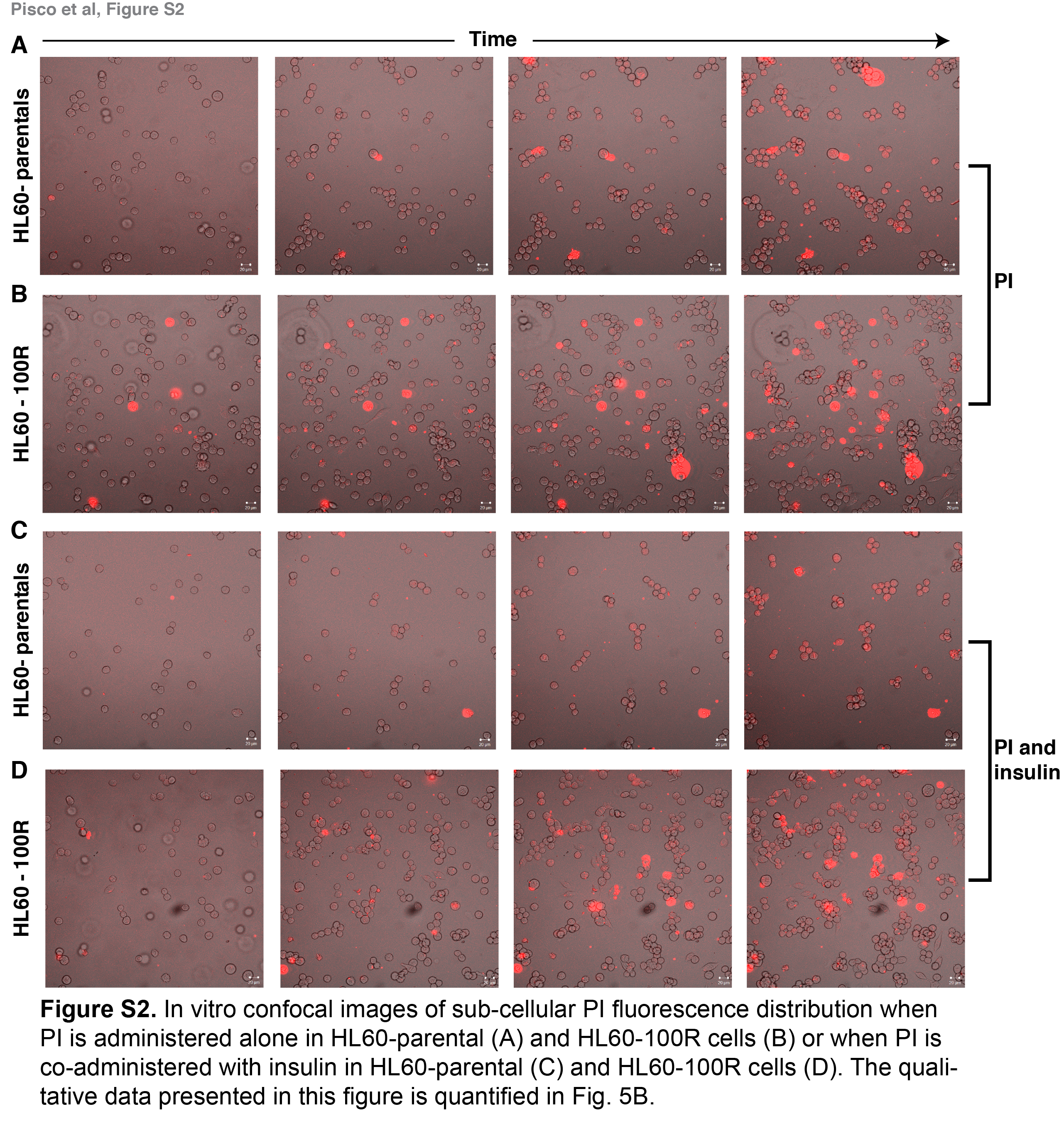

Supplement: Supplementary file 2 [file Image_2.TIF]
